# Resilience in Patients with Ischemic Heart Disease

**DOI:** 10.5935/abc.20160012

**Published:** 2016-02

**Authors:** Conceição Maria Martins de Lemos, David William Moraes, Lucia Campos Pellanda

**Affiliations:** 1 Instituto de Cardiologia do Rio Grande do Sul - Fundação Universitária de Cardiologia - IC/FUC - UFCSPA, Porto Alegre, RS - Brazil; 2 Universidade Federal de Ciências da Saúde de Porto Alegre - UFCSPA, Porto Alegre, RS - Brazil

**Keywords:** Resilience, Psychological, Myocardial Ischemia, Risk Factors, Psychosocial Impact.

## Abstract

**Background:**

Resilience is a psychosocial factor associated with clinical outcomes in chronic diseases. The relationship between this protective factor and certain diseases, such heart diseases, is still under-explored.

**Objective:**

The present study sought to investigate the frequency of resilience in individuals with ischemic heart disease.

**Method:**

This was a cross-sectional study with 133 patients of both genders, aged between 35 and 65 years, treated at Rio Grande do Sul Cardiology Institute - Cardiology University Foundation, with a diagnosis of ischemic heart disease during the study period. Sixty-seven patients had a history of acute myocardial infarction. The individuals were interviewed and evaluated by the Wagnild & Young resilience scale and a sociodemographic questionnaire.

**Results:**

Eighty-one percent of patients were classified as resilient according to the scale.

**Conclusion:**

In the sample studied, resilience was identified in high proportion among patients with ischemic heart disease.

## Introduction

Cardiac and vascular diseases are the main cause of death in the world, accounting for 31% of deaths, according to the World Health Organization (WHO).^1^ In 2012, 17.5 million out of 56 million of deaths were due to cardiovascular diseases.^2^

Studies from back in the 1960s have already pointed out that a multiplicity of factors are involved in the pathogenesis of cardiovascular diseases.^3,4^ However, psychosocial factors including depression, anxiety, social isolation, personality traits and stress have only recently been acknowledged by the literature.^5-8^

Resilience is a personality attribute which has been studied as a psychosocial factor related to the development of chronic diseases.^9^ In health sciences, it is defined as an individual's ability to deal with adversities without succumbing to them, and to surpass the negative effects of stressful life events.^10^ Resilience has been identified and reported in the context of several chronic diseases and medical conditions, including congenital heart diseases, diabetes, neurodegenerative diseases, human immunodeficiency virus (HIV) infection, medullary lesion, etc.

Strategies to increase resilience may be developed with therapeutic purposes aiming to improve patients' prognosis.^10^ Stress and resilience management also provide patients with comfort and well-being.^11^ Since the association between heart diseases and psychosocial factors has been well established,^6^ it is important to identify the resilient personality among cardiac patients. In this observational study, we aimed to identify resilience in patients with ischemic cardiomyopathy treated at Rio Grande do Sul Cardiology Institute, to contribute to the knowledge of psychosocial characteristics of this population. The close relationship between psychological aspects and the clinical disease is a privileged way of investigating the mechanisms of the development, prevention, diagnosis and treatment of ischemic cardiomyopathy.

## Methods

### Study design and ethical aspects

This was a cross-sectional study, conducted from March 2008 and July 2009 at Rio Grande do Sul Cardiology Institute, a center of reference in cardiology. The study was approved by the Ethics Committee of the hospital, and all participants signed a written informed consent.

### Subjects

A total of 133 individuals of both genders aged between 35 and 65 years were included in the study. Sixty-seven subjects were inpatients, randomly selected, with diagnosis of acute myocardial infarction (AMI) recorded in the medical records and established by the responsible cardiologist. For AMI diagnosis, the following criteria were considered: history of prolonged chest discomfort (>20 min) which was not relieved by sublingual nitrate, electrocardiographic changes consistent with necrosis (development of Q waves) and/or ST segment elevation > 1 mm, serial measurements of total creatine kinase (CK) and CK-MB fraction in the late phase. The other 66 participants were outpatients not diagnosed with AMI, which was confirmed by treadmill exercise testing, coronary angiography and ventriculography, recorded in their medical records.

### Assessment measures

For the assessment of resilience, we used a scale developed by Wagnild & Young12 and adapted in Brazil by Pesce et al.^13^ to measure levels of positive psychosocial adjustment to important life events. The final score was obtained by adding the score of each of the 25 questions which were rated from 1 ("totally disagree") to 7 ("totally agree"). The sum of the scores was divided by 175 and multiplied by 100. Subjects were then classified as "resilient" or "non-resilient", by using the mean and standard deviation criteria - one standard deviation was subtracted from the mean of the answers. Individuals with a score higher than this value were classified as resilient. Calculated mean and standard deviation were 84.67 and 8.47 respectively, and participants with a score higher than 76.2 were classified as resilient.

Data for the assessment of resilience were collected by a psychologist at the outpatient service of the Cardiology Institute, after the visit with the cardiologist. The sociodemographic form was analyzed by an investigator, blind to the cardiovascular diagnosis.

### Statistical analysis

Quantitative variables were described as mean and standard deviation, and qualitative variables were described as proportions. Between-group comparisons were performed by the chi-squared test and paired Student's t-test. Significance level was set at 5%. Data were analyzed by using the SPSS for Windows version 15.0.

## Results

**Figure 1:**
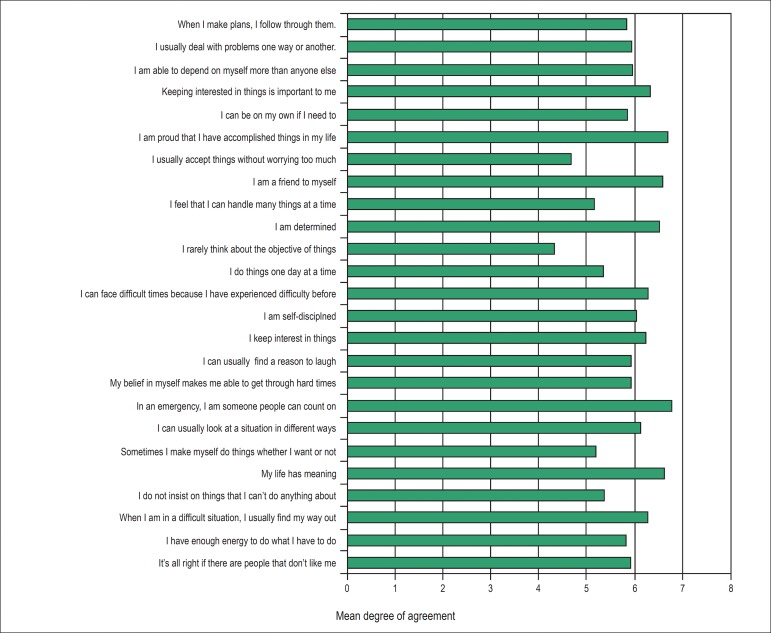
Mean rates of the answers to the questions proposed.

Demographic and clinical characteristics were compared between resilient and non-resilient patients (Table 1). Mean age of patients were 53.7 ± 8.4, and 81.2% of them were considered resilients. Figure 1 shows the mean scores of each of the 25 questions of the resilience scale.

**Table 1:** Comparison of demographic and clinical characteristics of patients with ischemic heart disease classified as "resilients" and "non-resilients"

	Total	Resilience		
	n = 133	n = 108 Yes^a^ (> 76.2)	n = 25 No^b^ (≤ 76.2)	p value
Male sex	68 (51.1)	58 (53.7)	10 (40.0)	0.217
Age, mean ± SD	53.7 ± 8.4	54.7 ± 7.9	49.4 ± 9.4	0.004
White race	109 (81.9)	87 (80.5)	22 (88.0)	0.383
Cohabitation	84 (63.1)	65 (60.1)	19 (76.0)	0.140
Number of years of education mean ± SD	7.8 ± 5.3	7.8 ± 4.7	7.8 ± 5.5	0.987
Smoking	45 (33.8)	31 (28.7)	14 (56.0)	0.009
Diabetes	34 (25.5)	28 (25.9)	6 (24.0)	0.842
Obesity	56 (42.1)	46 (42.5)	10 (40.0)	0.813
Sedentary lifestyle	50 (37.5)	39 (36.1)	11 (44.0)	0.463
Dislipidemia	46 (34,5)	36 (33,3)	10 (40,0)	0,528
Hipertensão	91 (68,4)	73 (67,5)	18 (72,0)	0,669
História familiar de IAM	73 (54,8)	58 (53,7)	15 (60,0)	0,569
Uso de medicação	109 (81,9)	90 (83,3)	19 (76,0)	0,390
Alcoolismo	23 (17,2)	19 (17,5)	4 (16,0)	0,835

a Individuals were classified as ‘resilient’ according to the assessment measures of this study.

b Individuals were classified as ‘non-resilient’ according to the assessment measures of this study

The percentage of patients classified as resilient was 74.2% among patients with previous diagnosis of AMI, and 88.1% among patients without the AMI diagnosis (p = 0,041).

## Discussion

In this cross-sectional study on patients with ischemic cardiomyopathy, we observed a high proportion of patients considered as resilient.

Chronic diseases are generally associated with long-term degenerative conditions that require continuous attention and adaptive behavior from patients and caregivers, in addition to access to all information needed for adequate management of the disease.^14^ For this reason, they represent a real adversity in patients' lives, by invoking resilience methods during the illness process.

A study on Duchenne muscular dystrophy reported that 84% of the studied children population was not found to be psychosocially at risk, but rather resilient.^15^ In a sample of 95 subjects with chronic pain, psychological resilience seemed to reduce pain catastrophizing events.^16^ Another study on 30 adolescents with type 1 diabetes suggested that the use of coping strategies was associated with indicators of resilience.^17^ The importance of resilience was also highlighted in a study on patients with HIV infection, due to peculiarities of the virus and HIV-related social stigma.^18^ A study involving 46 families of children with cardiac arrhythmia found a high level of resilience among the patients,^19^ and a cohort study investigating male adolescents suggested that low resilience to stress may be a risk factor for stroke.^20^

According to the literature, there is a great variety in how individuals react to adversity.^21,22^ On one end, there are those who are able to live through extremely adverse situations without significant sequelae, and mechanisms of this phenomenon have been investigated by studies on resilience.

Different life situations may have distinct meanings to people. Many theoretical models seek to characterize such subjectivity in defining adversity,^22,23^ since depending on the repertory of psychological capacities developed by the individual through his life, one single situation may be faced either as a challenge that motivates confrontation or an adversity that put him in a situation of frailty.

The concept of resilience depends on two basic assumptions:^24^ the occurrence of an adverse or stressing event throughout the individual's life, and the development of psychological mechanisms that allow for surpassing such potentially traumatic events. In the early studies on resilience over 40 years ago, it was believed that resilience was an innate attribute of some individuals, such as the "invulnerable children",^21^ mentioned by some authors. Such invulnerability would make these children able to deal with adverse situations. Lemos et al.^9^ point out the individual's subjective look at the level of exposure and individual limits in face of adversities, so that the same event may be faced as a danger by some individuals and as a challenge by others. This difference corresponds to one's resilience capacity.

However, there has been a change in the understanding of resilience during patients' clinical course. Although resilience was considered as an intrinsic, innate characteristic of an individual at first, it is currently considered as a dynamic process,^9,24^ gradually developed throughout the life span, by the facing and overcoming of adversities. Therefore, contributions of recent studies on the theme is that rather than a stable attribute that may be present or absent, resilience is displayed as a spectrum of individuals who are more or less resilient, subjected to continuous learning about the mechanisms of resilience. Thus, the degree of resilience of an individual would be temporally registered through his life course, and any attempt to measure this attribute would result in a mere snapshot.

We believe that one of the most relevant results of this study is that the subjects classified as more resilient were also the older patients. Such correlation is in accordance with the theoretical models on which resilience is based - considering the assumption that resilience is a dynamic, gradually developed phenomenon, it is expected that older individuals are also more resilient. However, one may note that this is a relative model, since a previous study^25^ has suggested that, although resilience is a dynamic event, it does not exhibit a linear progression, but rather, it is subjected to advances and retreats. Therefore, in absolute values, the robustness of resilience is not directly proportional to age.

It is not new to the medical literature^26^ the idea that aging is a risk factor for depression. This reminds us of the existence of multiple elements that, in conjunction with resilience, have a psychosocial effect on disease process. Aging, when associated with symptoms of depression, competes against resilience, and should be included in the analyses.

Possibly a legacy of the "invulnerable children" concept, many studies on resilience have been conducted with children. In light of the recent understanding of resilience as a construct of life, we believe that investigations on elderly patients, similar to what we propose here, will give a contribution to existing knowledge on the theme. Several studies have highlighted the association of resilience with chronic diseases, and it seems that the length of disease course and the age of studied population may yield significant correlation results.

One of our findings, difficult to be explained, is the negative association between resilience and smoking. Nearly one third of our population consisted of smokers, although more than half (56%) of non-resilient patients were smokers (p = 0.009). Beyond the hypothesis of a spurious association, this result suggests a wide range of possibilities to be investigated, from biochemical causes by a direct effect of tobacco, to psychosocial causes by the association of smoking with personality development, in addition to its ability to generate psychological defense mechanisms.

The study has some limitations. To our knowledge, there is no instrument available in the scientific literature designed to assess resilience specifically in cardiac patients. However, the resilience scale adopted in this study has been used in other groups of patients with chronic disease.^27-29^ In addition, there is no consensus on the cut-off points to classify individuals as "resilient" and "non-resilient". Any categorization may result in a simplistic definition, based on an arbitrary model, though needed until new studies present more satisfactory methods. From our standpoint, however, the usefulness of discriminating between resilient and non-resilient individuals lies more on its comparison with other clinical, behavioral and psychosocial variables, than on the label of "resilient" or "non-resilient" itself. These comparisons have the potential to clarify the mechanisms of resilience, and ultimately may lead to therapeutic approaches that promote this protective factor for positive clinical outcomes. Also, although the design of our study has met the objective of identifying resilience in the study population, one limitation was the fact that patients were evaluated at only one point at time. A prospective study investigating resilience and its correlation with cardiac disease over time would contribute to the knowledge about the mechanisms of resilience associated with the outcomes of chronic diseases.

## Conclusion

A high proportion of resilient individuals were identified among patients with myocardial infarction. Further studies are suggested to establish the relationship between resilience and the clinical outcome of patients over time, and to develop strategies to increase resilience in individuals experiencing adverse conditions.
